# Evaluating the quality and educational utility of YouTube videos in teaching human surface anatomy

**DOI:** 10.1002/ase.70160

**Published:** 2025-11-20

**Authors:** Anas J. Mistareehi, Ibrahim Hoja, Abdulrahman Alraddadi, Heba H. Ghozlan, Ayman Mustafa, Mohammed Z. Allouh

**Affiliations:** ^1^ Department of Anatomy, Faculty of Medicine Jordan University of Science and Technology Irbid Jordan; ^2^ Health Sciences, College of Medicine University of Saskatchewan Saskatoon Saskatchewan Canada; ^3^ Department of Basic Medical Sciences, College of Medicine King Saud Bin Abdulaziz University for Health Sciences (KSAU‐HS) Riyadh Saudi Arabia; ^4^ King Abdullah International Medical Research Center (KAIMRC) Ministry of National Guard, Health Affairs Riyadh Saudi Arabia; ^5^ Department of Physiology and Biochemistry, Faculty of Medicine Jordan University of Science and Technology Irbid Jordan; ^6^ Department of Basic Medical Sciences, College of Medicine, QU Health Qatar University Doha Qatar; ^7^ Department of Anatomy, College of Medicine and Health Sciences United Arab Emirates University Al Ain United Arab Emirates

**Keywords:** anatomy instruction, educational videos, medical education, surface anatomy, YouTube

## Abstract

YouTube is increasingly used by medical and health science students as a supplementary learning tool. However, the quality and educational value of surface anatomy videos on YouTube remain underexplored. This study aimed to systematically evaluate the quality, reliability, and educational usefulness of YouTube videos focusing on human surface anatomy. A structured YouTube search was conducted (December 2024–January 2025), targeting the seven primary body regions with specific keywords (e.g., “surface anatomy,” “bone landmarks,” and “dermatomes”). The top 30 videos per search term were selected. Two anatomists independently assessed each video using the Anatomy Content Score (ACS), Global Quality Scale (GQS), modified DISCERN (mDISCERN), and Journal of the American Medical Association (JAMA) benchmarks. Inter‐observer agreement was evaluated via Kappa coefficient. Associations between video quality scores and YouTube metrics (view count, like ratio, interaction index) were examined using nonparametric tests. Among 1050 retrieved videos, 85 (8%) met inclusion criteria; 48 (56.5%) were classified as “useful” (ACS ≥ 13, GQS ≥ 4). Longer video duration was significantly (*p* < 0.001) associated with higher usefulness, whereas view count, like ratio, and interaction index did not correlate with usefulness. ACS strongly correlated with GQS (*r_s_* = 0.754) and both correlated moderately with mDISCERN. No significant differences in video quality were observed across body regions, search rankings, presented material type, or upload period (pre‐ vs. post‐COVID‐19). YouTube offers a moderate‐quality resource for learning surface anatomy, with approximately 60% of evaluated videos deemed useful. Popularity metrics are unreliable indicators of video educational quality, underscoring the need for peer‐reviewed, high‐quality digital resources.

## INTRODUCTION

The global reliance on the Internet has accelerated across various aspects of daily life, becoming a cornerstone in scientific and educational domains. Many academic institutions have increasingly adopted online education through various internet‐based platforms. Integrating the Internet into education has become a fundamental aspect recognized across multiple levels.^1^, ^2^ The COVID‐19 pandemic significantly amplified the dependence on the Internet for education and the dissemination of information.^3^, ^4^ The educational content available online is vast and diverse, offering numerous formats such as web pages, platforms, and multimedia sites that enhance the accessibility of scientific information to students, sparking intrigue and engagement. YouTube, in particular, stands out as a significant platform for hosting educational videos, providing students with simplified or detailed explanations on specific topics.^5^

Surface anatomy, a fundamental branch of gross anatomy, focuses on identifying anatomical landmarks visible or palpable on the body's surface, providing a crucial link between external structures and internal anatomy.^6^, ^7^ Mastery of surface anatomy is essential in clinical practice, where techniques such as palpation, percussion, and auscultation rely on this knowledge for accurate diagnosis and procedures.^8^, ^9^, ^10^ Traditional methods of teaching surface anatomy, including cadaveric dissections, plastinated and plastic anatomical models, interactive learning, and textbooks, have both strengths and limitations in conveying the three‐dimensional and dynamic nature of anatomical structures, which are essential for clinical competence.^11^, ^12^ Recent innovations in medical education, including the development of assessment tools like free‐response short‐answer questions,^13^ have emphasized the importance of aligning teaching methods with modern evaluation strategies, further supporting the shift toward more interactive and visual learning approaches. Recent research also demonstrates that immersive virtual reality applications can significantly enhance undergraduate students' self‐directed learning competencies in anatomy education.^14^ Video‐based learning platforms, particularly YouTube, offer a valuable approach in anatomy education by improving spatial understanding through real‐time visualization and repetition of complex anatomy structures.^15^, ^16^

In 2012, Azer identified a significant gap in the availability of high‐quality YouTube videos dedicated to surface anatomy.^17^ The quality and educational value of online surface anatomy videos remain largely unexamined, despite the growing reliance on online databases, including YouTube, as sources of scientific information and medical education in recent years.^2^, ^18^ Concerns arise from students' strong preference for video‐based resources in anatomy education,^19^ and the notable lack of standardized frameworks to assess the reliability of YouTube's medical education content.^5^, ^18^

This study aimed to bridge this gap by systematically evaluating the quality of YouTube videos focused on surface anatomy. The study utilized validated assessment tools to evaluate content accuracy, pedagogical effectiveness, and alignment with competency‐based learning objectives. The study findings will provide insights into the strengths and limitations of YouTube as a resource for surface anatomy education, guiding both educators and students in effectively utilizing digital learning materials.

## MATERIALS AND METHODS

### Search strategy and video selection criteria

A structured YouTube search was conducted between December 25, 2024, and January 15, 2025, to extract educational videos focusing on surface anatomy for seven primary body regions: the Head and Neck, Upper Limb, Thorax, Abdomen, Pelvis and Perineum, Lower Limb, and the Back. For each of these body regions, we used specific keywords “surface anatomy,” “anatomy body painting,” “living anatomy,” “bone landmarks,” and “dermatomes” to capture a wide range of relevant video content for each region. Previous studies indicate that most YouTube users regularly scan the first 30 videos^20^, ^21^; therefore, the top 30 videos for each search term were included in the study. Videos unrelated to the subject, videos that lack audio or visuals, videos that describe research findings, videos in non‐English languages, and repeated videos were excluded from the study.

The search was conducted using a non‐logged‐in, incognito, cache‐cleared browser, preventing any algorithmic learning or personalization by YouTube and ensuring that no prior browsing history or personalized data influenced the results. The global YouTube website (https://www.youtube.com/?gl=US&hl=en) was used to conduct the search, avoiding any country‐specific filters or localization biases that might influence the results. We used the “Relevance” filter to sort search results, allowing us to observe the videos that YouTube deemed most pertinent to the keywords under default settings. No additional filters were applied to modify the search results, ensuring our findings reflected the platform's standard operation for an unregistered user.

### Evaluation of video visibility

To analyze video visibility in search results, videos from each keyword search were categorized into three ranked groups based on their position: top 1–10, 11–20, and 21–30. The like ratio and view rate for evaluated YouTube videos were calculated using specific formulas. The like ratio was determined by the formula: [(Number of Likes × 100)/(Number of Likes + Number of Dislikes)]. The view rate is calculated by dividing the number of views by the number of days since upload. Additionally, the Interaction Index assesses viewer engagement with the videos using the formula: [((Number of Likes − Number of Dislikes)/Total Number of Views) × 100]. To determine whether a video was uploaded before or after COVID‐19, videos uploaded in April 2020 or later were classified as “after COVID,” while those uploaded before this date were categorized as “before COVID.” In addition, the videos were categorized based on demonstration materials into drawings/images, models, and living/cadaver demonstrations to analyze the presentation methods used in the videos.

### Evaluation of videos

Two anatomists independently analyzed the selected videos. The following information was recorded: the video's duration, upload date, upload source, country of origin, authorship, view count, and number of likes and dislikes.

To evaluate the videos' effectiveness in delivering sufficient surface anatomy information, we employed the Anatomy Content Score (ACS), a useful score developed by Azer in 2012.^17^ Briefly, there are 5 major and 6 minor criteria. Two points and 1 point were given for each item in the major and minor criteria, respectively (Table 1). An allocated score was given for each fulfilled item, while unfulfilled items received a zero. No partial scores were assigned. Videos scoring 13 or higher are regarded as useful (meeting all major criteria is mandatory). Additionally, the Global Quality Scale (GQS), a 5‐point rating system (Appendix 1, Table S1), was used to assess the quality and flow of information presented in the videos. Videos are considered useful if they score 4 points or higher, while those scoring 3 points or lower are classified as not useful. Furthermore, the modified DISCERN (mDISCERN) score and Journal of the American Medical Association (JAMA) criteria were applied to rate information reliability and assess source transparency and accountability of the videos, respectively (Appendix 1, Tables S2 and S3).

**TABLE 1:** Anatomy Content Score (ACS) assessment criteria.

Major criteria
Contents are scientifically correct
Images are clear
Creator and/or organization mentioned
Topic is clearly presented
The video uses living bodies, models, drawings to explain difficult issues
Minor criteria
Covers topic identified
Designed at the level of undergraduate medical/health sciences students
Sounds are clear and background is free from noises
Time to download is reasonable
Information about the creator is up‐to‐date
Educational objectives are stated

*Note*: Videos were assessed based on five major criteria (2 points each) and six minor criteria (1 point each). Each fulfilled criterion received the allocated score, while unfulfilled items scored zero. A video was considered useful if it scored 13 or higher, with all major criteria met.

### Statistical analysis

Cohen's Kappa coefficient was used to assess the inter‐observer agreement for the four quality scoring systems. The IBM SPSS Statistics software (standard version 29.0.0.0, IBM, Armonk, NY, USA). was used for data analysis. The Shapiro–Wilk test showed that the data were not normally distributed. Therefore, the nonparametric Mann–Whitney *U* test was used to compare the numerical data. A Chi‐square test was used to analyze the categorical data. An independent sample Kruskal–Wallis test followed by pairwise comparisons was conducted for three or more independent groups analysis. Spearman's rank correlation coefficient (*r_s_*) was used to evaluate the correlation between video quality measures and YouTube metrics. Statistical results were considered significant when *p*‐values were < 0.05.

## RESULTS

**FIGURE 1:**
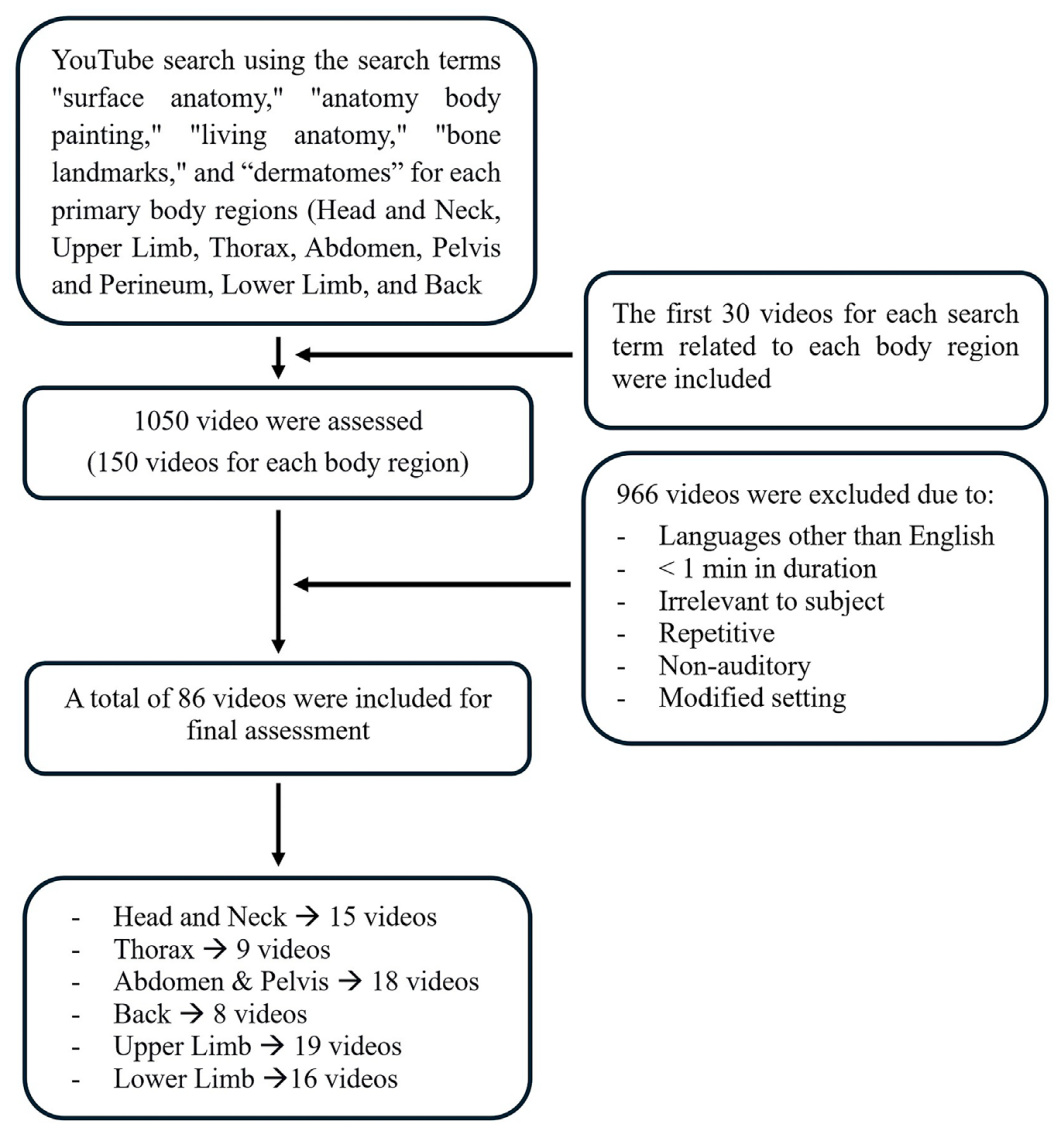
Flowchart depicting the YouTube search strategy with inclusion and exclusion criteria.

Out of the first 1050 videos retrieved through keyword searches, 85 videos (8%) met the inclusion criteria and were analyzed. The distribution of videos across anatomical regions was as follows: head and neck (*n* = 15, 17.6%), thorax (*n* = 9, 10.6%), abdomen (*n* = 18, 21.2%), back (*n* = 8, 9.4%), pelvis and perineum (*n* = 1, 1.2%), upper limb (*n* = 19, 22.4%), and lower limb (*n* = 15, 17.6%). Due to the limited number of videos in the pelvis and perineum category (*n* = 1), this video was included in the abdomen group for further analysis (Figure 1). The Kappa coefficient demonstrated an almost perfect level of agreement among observers (ACS = 0.945, GQS = 0.880, mDISCERN = 0.821, and JAMA = 0.977). Any discrepancies in scoring were resolved through a collaborative review of the relevant videos. Further details about the videos are provided in Appendix 2.

### Basic characteristics of the videos

**FIGURE 2:**
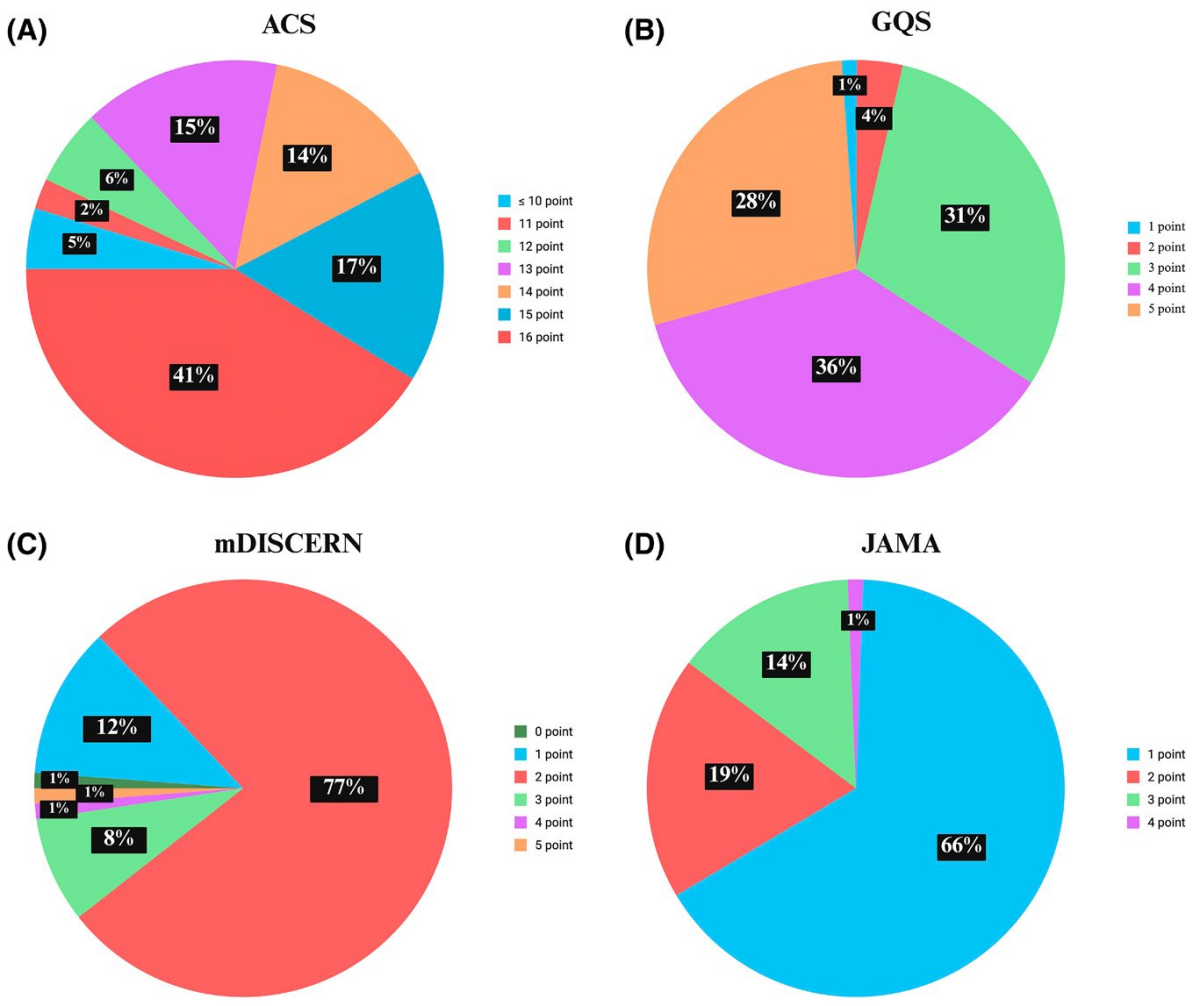
The distribution of YouTube video quality scores across the four key systems for evaluating YouTube videos. (A) Anatomy Content Score (ACS), (B) Global Quality Scale (GQS), (C) modified DISCERN (mDISCERN), and (D) Journal of the American Medical Association (JAMA) criteria. Each chart displays the percentage (rounded to the nearest integer) of videos assigned different scores for each metric. The ACS evaluates the comprehensiveness of anatomical content, the GQS assesses overall video quality, the mDISCERN measures reliability, and the JAMA criteria examine source credibility. Created in http://BioRender.com.

The descriptive statistics for the analyzed video metrics and quality assessment scores are presented in Table 2. A total of 85 videos were analyzed, with a wide range in duration, spanning from 82 to 5650 s (mean video duration = 794.1 s). The view count varied considerably across videos, ranging from 290 to 1,063,133 views. The view ratio (views per day) had a large range (0.1 to 3375). Regarding user engagement, the number of likes ranged from 5 to 6991; dislikes ranged from 0 to 269. The like ratio was consistently high, ranging from 88% to 100%. The interaction index, reflecting user engagement, ranged from 0.3 to 9.5. Videos were uploaded ranging from 185 to 4941 days. Regarding the quality and reliability assessment scores, the ACS scores ranged from 7 to 16 points (mean 14 points). Specifically, 41% of videos scored 16 points, 17% scored 15 points, 14% scored 14 points, and 15% scored 13 points, indicating a tendency toward moderate to high anatomical content completeness. The GQS scores ranged from 1 to 5 points (mean 3.9), with most videos scoring 4 points (36%) or 5 points (28%), followed by 3 points (31%), 2 points (4%), and a small proportion scoring 1 point (1%). The mDISCERN scores showed a wide distribution from 0 to 5 points (mean 2.0), with a predominant clustering at 2 points (77%), followed by 1 point (12%) and 3 points (8%). Additionally, scores of 0, 4, and 5 points each accounted for 1% of the videos. This pattern suggests that most videos had moderate reliability, with relatively few achieving very low or high mDISCERN scores. Lastly, JAMA scores ranged from 1 to 4 points (mean 1.5), with two‐thirds of videos (66%) scoring 1 point, 19% scoring 2 points, 14% scoring 3, and 1% scoring 4 points, highlighting generally low to moderate source credibility across videos (Figure 2).

**TABLE 2:** Descriptive statistics of evaluated videos (*n* = 85).

	Min	Max	Mean (±SD)	Median
Duration (s)	82	5650	794.1 (874.0)	521
View	290	1,063,133	66,122.5 (142,992.6)	20,022
View ratio	0.1	3375	68.1 (365.8)	8
Like	5	6991	831.5 (1433.5)	240
Dislike	0	269	19.6 (38.9)	4
Like ratio	88	100	98.3 (2.3)	99
Interaction index	0.3	9.5	1.8 (1.2)	1.5
Days since upload	185	4941	1962 (1138.8)	1657
Search ranking	1	3	1.66 (0.8)	1
ACS	7	16	14 (1.9)	15
GQS	1	5	3.9 (0.9)	4
mDISCERN	0	5	2.0 (0.4)	2
JAMA	1	4	1.5 (1)	1

*Note*: Key metrics for the 85 analyzed videos including videos duration, engagement metrics, search ranking, and educational quality assessment scores (ACS, Anatomy Content Score; GQS, Global Quality Scale; mDISCERN, modified DISCERN; JAMA, Journal of the American Medical Association). Data are presented as minimum, maximum, mean (±SD), and median values.

Table 3 provides the descriptive statistics for the video metrics and quality assessment scores across different regions. While view count varied substantially across regions, with abdomen and pelvis videos receiving the highest mean views (139,529.3) and head and neck videos receiving the lowest (18,420.1), the differences were not statistically significant (*p* = 0.234). Similarly, view ratio, like count, dislike count, interaction index, and like ratio showed variations across anatomical regions but did not reach statistical significance. Video duration was the only parameter that showed a statistically significant difference between anatomical regions (*p* = 0.003), with videos related to the head and neck having the longest mean duration (876.1), whereas videos covering the back had the shortest mean duration (229.6). However, post hoc analysis indicated that the video duration of the back region was significantly different from both the abdominal and upper limb regions (*p* < 0.001, Bonferroni‐adjusted *p* < 0.0033). When assessing video quality, ACS, GQS, mDISCERN, and JAMA scores, the Kruskal–Wallis test showed no statistically significant differences across body regions (*p* = 0.179, 0.238, 0.105, and 0.477, respectively), indicating that the educational and informational value of the videos remained comparable regardless of the anatomical region discussed. Additional details on the descriptive analysis of the videos categorized by anatomical regions are provided in Table 3.

**TABLE 3:** Descriptive statistics of evaluated videos by anatomical regions.

	Head and neck		Thorax		Abdomen and pelvis		Back		Upper limb		Lower limb		*p*‐value
	15		9		19		8		19		15		
	Mean (±SD)	Median	Mean (±SD)	Median	Mean (±SD)	Median	Mean (±SD)	Median	Mean (±SD)	Median	Mean (±SD)	Median	
Duration (s)	876.1 (1118.7)	435	644.9 (485.4)	521	706.2 (433.5)	604	229.6 (105.2)	231.5	657.2 (1302.7)	910	592.1 (482.5)	516	0.003
View	18,420.1 (23,447.6)	5009	74,998.8 (111,867.0)	39,287	139,529.3 (263,250.3)	27,732	38,021.3 (57,961.3)	6155.5	63,168.7 (92,176.3)	25,070	34,246.3 (43,843.6)	7942	0.234
View ratio	9.92 (10.4)	3.9	27.0 (23.9)	25.4	223.4 (766.1)	17.8	15.9 (26.1)	2.8	39.5 (55.2)	12.2	18.0 (27.2)	4.7	0.160
Like	231 (236.0)	116	1100.8 (1549.4)	906	1446.47 (2119.2)	684	478 (923.1)	111.5	1017.6 (1537.2)	296	444.53 (662.1)	107	0.111
Dislike	7.9 (15.1)	0	7.8 (36.2)	13	33.5 (65.7)	4	13.9 (26.0)	2.5	20.1 (31.1)	6	10.4 (19.8)	2	0.409
Like ratio	98.2 (3.2)	100	97.7 (2.6)	98.1	98.8 (1.4)	99.5	97.7 (3.5)	99.5	98.0 (2.2)	98.4	98.7 (1.3)	99.3	0.772
Interaction index	2.1 (2.1)	1.4	1.8 (0.8)	1.6	1.9 (1.1)	1.7	1.8 (1.4)	1.8	1.7 (1.0)	1.5	1.1 (0.7)	1.3	0.818
Days since upload	1549.3 (888.5)	1281	2194.1 (1297.0)	1514	1758.1 (1154.0)	1415	2220.3 (1187.6)	1761	2105.3 (1263.5)	1883	2175.7 (1082.8)	1737	0.463
Search ranking	1.7 (0.9)	1	1.6 (0.7)	1	1.8 (0.9)	2	2 (0.8)	2	1.4 (0.6)	1	1.7 (0.8)	1	0.421
ACS	13.4 (2.7)	15	14.8 (1.6)	16	13.5 (1.9)	15	13.5 (2.7)	13	15.1 (1.1)	16	14.7 (1.3)	15	0.179
GQS	3.9 (1.2)	4	3.9 (1.1)	4	3.8 (0.7)	4	3.4 (0.7)	3.5	4.2 (0.9)	4	3.8 (0.8)	4	0.238
mDISCERN	1.7 (0.7)	2	2 (0.5)	2	2.26 (0.8)	2	1.6 (0.5)	2	2.1 (0.5)	2	2 (0.5)	2	0.105
JAMA	1.5 (0.8)	1	1.9 (0.9)	2	1.14 (0.7)	1	1.3 (0.5)	1	1.6 (09)	1	1.3 (0.7)	1	0.477

*Note*: Comparative analysis of videos duration, engagement metrics, search ranking, and quality assessment scores (ACS, Anatomy Content Score; GQS, Global Quality Scale; mDISCERN, modified DISCERN; JAMA, Journal of the American Medical Association) across six anatomical regions. Significant regional variation observed in video duration (*p* = 0.003). Data are presented as mean (±SD) and median values. Statistical significance (*p* < 0.05) was calculated using the Kruskal–Wallis test with pairwise comparisons.

### Correlation analysis

The correlation analysis revealed significant associations between various measures of video quality and YouTube metrics (Table 4). Notably, a strong positive correlation was observed between the ACS and GQS (*r*_*s*_ = 0.754, 95% CI [0.640, 0.835], *p* < 0.001), indicating that videos scoring high on ACS criteria also tend to exhibit higher overall quality as measured by GQS. Both ACS and GQS demonstrated moderate positive correlations with the mDISCERN score (ACS: *r*_*s*_ = 0.506, 95% CI [0.323, 0.653], *p* < 0.001; GQS: *r*_*s*_ = 0.525, 95% CI [0.345, 0.667], *p* < 0.001), further supporting the convergent validity of these quality assessment measures. Interestingly, ACS showed a weak to moderate positive correlation with the JAMA criteria (*r*_*s*_ = 0.323, 95% CI [0.112, 0.506], *p* < 0.01), while GQS exhibited a weaker but still statistically significant correlation (*r*_*s*_ = 0.219, 95% CI [−0.001, 0.418], *p* < 0.05). These correlational patterns suggest that greater source transparency is associated with only small improvements in content and educational quality, indicating that transparency is complementary to, but not a substitute for, content‐focused metrics such as ACS and educational utility ratings, such as GQS. Regarding video characteristics, ACS and GQS showed a moderate positive correlation with video duration (ACS: *r*_*s*_ = 0.414, 95% CI [0.214, 0.580], *p* < 0.001; GQS: *r*_*s*_ = 0.385, 95% CI [0.181, 0.557], *p* < 0.001), and mDISCERN was similarly correlated with video duration (*r*_*s*_ = 0.432, 95% CI [0.235, 0.595], *p* < 0.001), suggesting that longer videos tended to be of higher quality. However, neither ACS nor GQS showed significant correlations with view count (ACS: *r*_*s*_ = −0.006, 95% CI [−0.225, 0.214]; GQS: *r*_*s*_ = −0.021, 95% CI [−0.239, 0.199]), or view ratio (ACS: *r*_*s*_ = 0.061, 95% CI [−0.161, 0.276]; GQS: *r*_*s*_ = 0.047, 95% CI [−0.340, 0.079]), indicating that video quality, as measured by these tools, does not necessarily translate to higher viewership. The like ratio showed weak positive but nonsignificant correlations with ACS (*r*_*s*_ = 0.150, 95% CI [−0.071, 0.358]) and GQS (*r*_*s*_ = 0.184, 95% CI [−0.037, 0.288]), and a statistically significant negative correlation with JAMA (*r*_*s*_ = −0.225, 95% CI [−0.423, −0.006], *p* < 0.05). The interaction index correlated positively but weakly and nonsignificantly with ACS (*r*_*s*_ = 0.179, 95% CI [−0.042, 0.383]) and GQS (*r*_*s*_ = 0.186, 95% CI [−0.035, 0.389]), suggesting viewer engagement metrics may not reliably indicate video quality. Days since upload were not significantly correlated with ACS (*r*_*s*_ = −0.054, 95% CI [−0.270, 0.167]) or GQS (*r*_*s*_ = −0.102, 95% CI [−0.314, 0.120]), but showed a significant positive correlation with JAMA score (*r*_*s*_ = 0.295, 95% CI [0.081, 0.483], *p* < 0.01), implying that earlier uploaded videos might be associated with higher JAMA scores. Finally, search ranking exhibited weak but significant positive correlations with ACS (*r*_*s*_ = 0.221, 95% CI [0.002, 0.420], *p* < 0.05), GQS (*r*_*s*_ = 0.216, 95% CI [−0.003, 0.416], *p* < 0.05), mDISCERN (*r*_*s*_ = 0.269, 95% CI [0.053, 0.461], *p* < 0.05), and JAMA (*r*_*s*_ = 0.296, 95% CI [0.082, 0.484], *p* < 0.01), suggesting that higher‐quality videos are somewhat more likely to appear earlier in YouTube search results. Overall, these findings support the convergent validity and relevance of the quality assessment measures, while highlighting that popular engagement metrics such as views and like ratios do not necessarily reflect video quality.

**TABLE 4:** Correlation analysis of medical educational video quality scores (ACS, GQS, mDISCERN, JAMA) with YouTube metrics.

		ACS	GQS	mDISCERN	JAMA	Duration	View	View ratio	Like ratio	Interaction index	Days since upload	Search ranking
ACS	*r*_*s*_ 95% CI	1										
GQS	*r*_*s*_ 95% CI	0.754*** 0.640–0.835	1									
mDISCERN	*r*_*s*_ 95% CI	0.506*** 0.323–0.653	0.525*** 0.345–0.667	1								
JAMA	*r*_*s*_ 95% CI	0.323** 0.112–0.506	0.219* −0.001 to 0.418	0.328** 0.118–0.511	1							
Duration	*r*_*s*_ 95% CI	0.414*** 0.214–0.580	0.385*** 0.181–0.557	0.432*** 0.235–0.595	0.120 −0.102 to 0.331	1						
View	*r*_*s*_ 95% CI	−0.006 −0.225 to 0.214	−0.021 −0.239 to 0.199	0.092 −0.129 to 0.306	0.216* 0.047–0.003	0.027 −0.193 to 0.245	1					
View ratio	*r*_*s*_ 95% CI	0.061 −0.161 to 0.276	0.047 −0.340 to 0.079	0.164 −0.058 to 0.370	0.168 −0.053 to 0.374	0.138 −0.084 to 0.347	0.938*** 0.906–0.960	1				
Like ratio	*r*_*s*_ 95% CI	0.150 −0.071 to 0.358	0.184 −0.037 to 0.288	0.211 −0.009 to 0.411	−0.225* −0.423 to 0.006	0.089 −0.133 to 0.302	−0.674*** −0.778 to 0.534	−0.527 0.669–0.348	1			
Interaction index	*r*_*s*_ 95% CI	0.179 −0.042 to 0.383	0.186 −0.035 to 0.389	0.104 −0.118 to 0.316	−0.137 −0.346 to 0.085	0.306** 0.093–0.492	−0.361*** −0.538 to 0.154	−0.190 −0393 to 0.030	0.486*** 0.299–0.637	1		
Days since upload	*r*_*s*_ 95% CI	−0.054 −0.270 to 0.167	−0.102 −0.314 to 0.120	−0.101 −0.313 to 0.121	0.295** 0.081–0.483	−0.266* −0.459 to 0.050	0.338** 0.128–0.518	0.048 −0.173 to 0.265	−0.531*** −0.672 to 0.352	−0.497*** −0.646 to 0.311	1	
Search ranking	*r*_*s*_ 95% CI	0.221* 0.002–0.420	0.216* −0.003 to 0.416	0.269* 0.053–0.461	0.296** 0.082–0.484	0.316** 0.104–0.501	0.372*** 0.166–0.547	0.444*** 0.249–0.604	−0.245* −0.440 to 0.027	−0.078 −0.292 to 0.144	−0.014 −0.205 to 0.233	1

*Note*: Spearman correlation coefficients of educational quality scores (ACS, Anatomy Content Score; GQS, Global Quality Scale; mDISCERN, modified DISCERN; JAMA, Journal of the American Medical Association), and YouTube‐related variables, including video duration, views, view ratio, like ratio, interaction index, days since upload, and search ranking. Significant correlations marked as: *p < 0.05, **p < 0.01, and ***p < 0.001. Note the strong positive correlation between ACS and GQS scores versus weak correlations of ACS and GQS with popularity metrics (view count, view ratio, and like ratio).

### Evaluation of video usefulness

**FIGURE 3:**
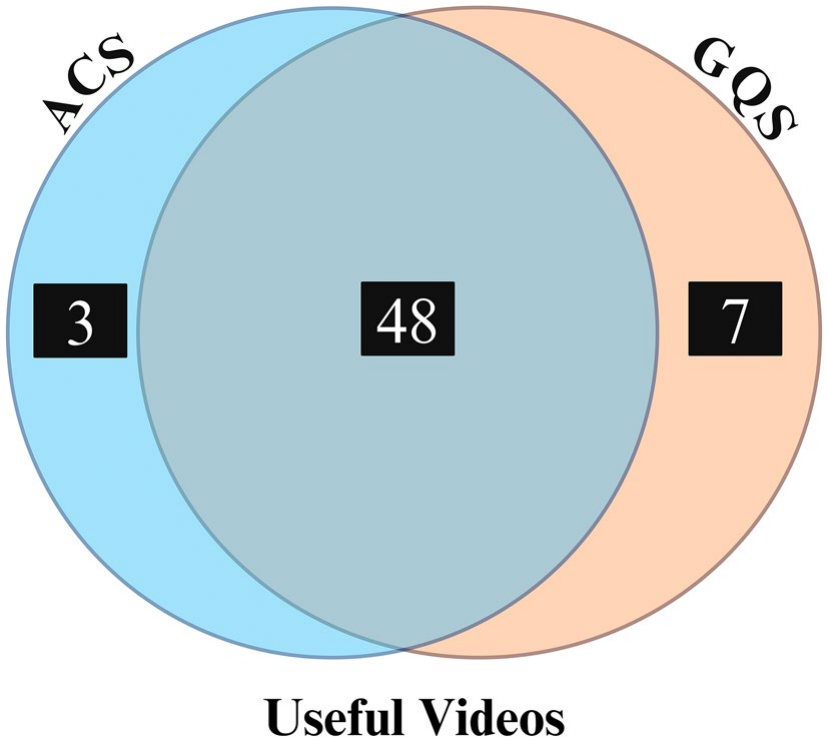
Useful videos identified by ACS and GQS. The overlap between videos classified as useful by the Anatomy Content Score (ACS) and the Global Quality Scale (GQS) systems. According to the ACS criteria, 51 videos were classified as useful, while the GQS criteria identified 55 useful videos. The assessment showed that 48 videos were classified as useful by both systems, with ACS and GQS independently classifying 3 and 7 additional videos as useful, respectively.

When we evaluated the usefulness of videos, we found that the ACS criteria recognized 51 videos as useful and 34 as not useful, while the GQS criteria identified 55 videos as useful and 30 as not useful. Notably, 48 videos were considered useful by both scoring systems (Figure 3). In terms of video characteristics, video length was significantly associated with usefulness, with useful videos being considerably longer than non‐useful videos (*p* < 0.001, Mann–Whitney *U* test). This suggests that longer videos may provide more comprehensive content, enhancing their educational value. However, other metrics, including the number of views, view ratio, likes, dislikes, like ratio, interaction index, and days since upload, did not show statistically significant differences between useful and non‐useful videos (*p* > 0.05 for all comparisons, Mann–Whitney *U* test) (Table 5).

**TABLE 5:** Comparative analysis of YouTube video metrics between useful and not useful educational content: ACS and GQS classifications.

	ACS					GQS				
	Useful 51		Not useful 34		*p*‐value	Useful 55		Not useful 30		*p*‐value
	Mean (±SD)	Median	Mean (±SD)	Median		Mean (±SD)	Median	Mean (±SD)	Median	
Video length	1030.5 (1032.1)	754	439.4 (337.9)	297	<0.001	726 (1013.0)	736	415.4 (269.4)	317	<0.001
Date upload	1909.6 (1174.0)	1679	2041.0 (1096.6)	1631	0.622	1871 (1125.6)	1657	2139 (1163.1)	1668	0.351
Views	56,032 (91,551.7)	19,319	81,257.5 (197,336.9)	26,493	0.957	58,741.6 (100,318.3)	17,532	79,654.4 (200,482.6)	27,347	0.847
View ratio	30.9 (49.0)	8.8	123.8 (575.9)	6.7	0.578	31.4 (48.9)	8.8	135.3 (613.1)	7.1	0.666
Like	844.3 (1421.8)	290	812.6 (1472.1)	193.5	0.663	871.5 (1455.4)	290	758.3 (1414.1)	210	0.762
Dislike	14.2 (26.4)	2	27.6 (52.8)	6	0.315	18.0 (42.3)	2	22.5 (32.3)	6.5	0.215
Like ratio	98.7 (1.5)	99.5	97.6 (3.0)	98.0	0.169	98.7 (1.6)	98.5	97.5 (3.0)	98.5	0.091
Interaction index	2.0 (1.4)	1.6	1.5 (0.7)	1.4	0.101	2.0 (1.4)	1.6	1.5 (0.8)	1.4	0.089
Days since upload	1909.6 (1174.0)	1679	2041 (1096.5)	1631	0.622	1871 (1125.6)	1657	2129 (1163.1)	1668	0.351

*Note*: Comparative analysis of YouTube videos metrics (duration and engagement metrics) between Useful and Not Useful educational content, as classified by ACS (Anatomy Content Score, Score ≥ 13, should fulfill all major criteria) and GQS (Global Quality Scale, Score ≥ 4), was performed using the Mann–Whitney *U* test. Significant differences were observed only in video length (*p* < 0.001), with useful videos being substantially longer. Data are presented as mean ± standard deviation and median.

When evaluating the usefulness of videos based on different factors such as body region, search ranking, material used, and upload date relative to the COVID‐19 pandemic, no significant differences were observed in either the ACS or GQS criteria, as determined by the Chi‐square test. In terms of the body region, videos covering various anatomical regions did not show significant differences in usefulness (ACS: *p* = 0.699, GQS: *p* = 0.697). Similarly, when categorizing videos by search ranking (1–10, 10–20, 20–30), there were no statistically significant differences between useful and not useful videos in both the ACS and GQS criteria (ACS: *p* = 0.083, GQS: *p* = 0.078). Regarding the material used in the videos, including drawings, models, and the human body, no significant differences were found in terms of usefulness (ACS: *p* = 0.303, GQS: *p* = 0.155). Finally, when comparing videos based on the time frame of COVID (defining post‐COVID videos as those uploaded after March 2020), no significant differences in usefulness were found in either criterion (ACS: *p* = 0.530, GQS: *p* = 0.893) Table 6.

**TABLE 6:** Comparative analysis of YouTube video characteristics between useful and not useful educational content: ACS and GQS classifications.

	Total	ACS			GQS		
		Useful 51	Not useful 34	*p*‐value	Useful 55	Not useful 30	*p*‐value
Body region				0.699			0.697
Head and neck	15	8	7		10	5	
Thorax	9	6	3		6	3	
Abdomen and pelvis	19	11	8		11	8	
Back	8	3	5		4	4	
Upper limb	19	13	6		15	4	
Lower limb	15	10	5		9	6	
Search ranking				0.083			0.078
1–10	45	32	13		34	11	
10–20	24	11	13		12	12	
20–30	16	8	8		9	7	
Material used				0.303			0.155
Drawings/images	42	28	14		31	11	
Models	4	3	1		3	1	
Living/cadavers	39	20	19		21	18	
COVID				0.530			0.893
Before	36	23	13		23	13	
After	49	28	21		32	17	

*Note*: Comparison of YouTube video characteristics (body region, search ranking, material used, COVID‐19 pandemic era) by educational usefulness, based on ACS and GQS classifications, showed a trend toward higher usefulness in top‐ranked videos and reduced utility in cadaver/living specimen content, although differences were not statistically significant. No significant differences were observed across body regions or pandemic eras. Statistical significance was determined using the Chi‐square test, with *p*‐values reported for each comparison.

## DISCUSSION

This study evaluated the educational value and overall quality of YouTube videos related to surface anatomy, aiming to offer a guideline framework that helps students identify reliable learning resources while also supporting educators in the development of clear, engaging, and pedagogically effective videos. While prior investigations have analyzed YouTube videos for various anatomical education topics,^15^, ^16^, ^17^ this is the first systematic assessment of surface anatomy videos in depth for all body regions. The analysis revealed moderate overall usefulness of surface anatomy‐related YouTube videos, with only a subset of videos (51 and 55 out of 85 useful videos by ACS and GQS criteria, respectively) providing comprehensive coverage of anatomical landmarks and their clinical correlations.

YouTube has emerged as a prominent educational resource due to its open‐access platform, allowing users to freely view and share content.^18^ While YouTube holds potential as a supplementary instructional tool,^22^ concerns persist regarding the reliability of its medical content, as videos are not peer‐reviewed prior to publication.^18^, ^23^ However, a recent study revealed that 93.2% of male and 89.3% of female medical students relied on YouTube for anatomy learning.^24^ Interestingly, recent studies further emphasized the growing role of innovative educational methods in anatomy teaching, including video‐assisted, three‐dimensional (3D) applications, and narrative anatomy education. The findings indicate that while traditional dissection remains optimal, 3D and video‐assisted methods serve as effective alternatives, enhancing comprehension through interactive and visual learning. Moreover, narrative approaches integrating storytelling and descriptions have been shown to improve students' engagement, understanding, and retention of anatomical concepts. These advancements align with the increased use of online video platforms like YouTube for anatomy education, highlighting the potential for well‐designed digital content to complement or even enhance traditional teaching modalities in surface anatomy learning.^25^, ^26^

Surface anatomy, a cornerstone of clinical practice for procedures such as physical examinations and landmark identification, is among the most sought‐after topics for video‐based learning. However, the variable quality of surface anatomy YouTube videos raises critical questions about their suitability for professional education. In the current study, from an initial pool of 1050 videos retrieved using relevant keywords on the YouTube platform, only 85 videos met the inclusion criteria. This finding underscores the importance of thoughtful keyword selection by students when searching for educational content and highlights the need for educators to use precise and consistent terminology when uploading instructional videos to enhance their discoverability and educational value. Among the 85 videos that met the inclusion criteria, approximately 60% were considered useful based on established evaluation standards. This proportion is notably higher than what has been reported in previous studies, where only about 25% of analyzed YouTube videos on skull and heart anatomy were useful.^15^, ^16^ Similarly, in 2012, Azer found that only 27% of the videos were useful.^17^ This discrepancy may be attributed to the stricter inclusion criteria applied in the current study, which filtered out a significant number of low‐quality or irrelevant videos. Additionally, the higher proportion of useful videos may be explained by the presence of specific content creators (uploaders) who contributed a series of high‐quality videos that covered almost all body regions, thereby increasing the overall percentage of educationally valuable content.

A noteworthy observation from our findings is the striking imbalance in coverage of certain body regions, particularly the pelvis and perineum. Only one qualifying video on this region was identified, prompting its merger with the abdomen category for analysis. This low representation could stem from a range of factors, including cultural sensitivities surrounding the pelvic area, the shortage of specialized expertise to create instructional materials on this region, or simply lower demand from viewers (students). Irrespective of the cause, this gap carries pedagogical implications. Surface anatomy of the pelvis and perineum is essential for numerous clinical procedures.^27^ However, students relying heavily on YouTube might struggle to locate sufficient, high‐quality content on this body region. Recognizing such discrepancies highlights the importance of verifying the completeness of any video collection used for teaching and ensures that learners receive a balanced educational experience across all major anatomical regions.

Following the World Health Organization's declaration of COVID‐19 as a global pandemic in March 2020, many academic institutions transitioned abruptly to fully online teaching formats. This shift significantly increased the demand for and reliance on online educational resources, including platforms such as YouTube.^28^ In the present study, we compared the usefulness of videos uploaded before and after the pandemic to explore whether this shift influenced the quality of surface anatomy videos. Of the 85 videos included in our analysis, 36 (42%) were uploaded before the onset of the pandemic, while 49 (58%) were uploaded afterward. Despite the increased educational demands during the pandemic and the growing awareness of digital content creation, our findings revealed no statistically significant differences in the usefulness scores between the two periods. This suggests that, while the quantity of educational videos may have increased post‐pandemic, the overall quality in terms of educational value remained consistent. This finding indicates that while educators and institutions increasingly adopted YouTube as an educational tool during the pandemic, the quality and educational effectiveness of video content did not notably improve during that period.

The analyzed videos collectively garnered approximately 5.5 million views, with a mean of 66,122.5 views per video. However, no statistically significant difference in view counts was observed between useful and non‐useful videos. This aligns with studies demonstrating that the number of viewers was poorly correlated with educational quality.^15^, ^21^ This finding may be explained by the way YouTube's search and recommendation system operates. In 2015, Madathil suggested that the retrieval and visibility of health‐related videos on YouTube are heavily influenced by keyword matching rather than by content quality or educational rigor.^29^ As a result, videos with popular or trending keywords may receive disproportionately high view counts regardless of their accuracy or instructional value. Importantly, our results showed that useful videos were statistically significantly longer in duration compared to non‐useful videos. This finding is consistent with previous studies, which have indicated that scientifically useful YouTube videos tend to be longer in duration.^15^, ^30^ A possible explanation for this observation is that comprehensive anatomical explanations require extended durations to adequately address complex spatial relationships, detailed structural descriptions, and relevant clinical applications. Shorter videos may lack the depth necessary to convey such multifaceted content, thereby reducing their overall educational value. However, it is important to note that longer videos must be structured effectively to maintain learner engagement. Brame^31^ argued that while brevity can improve viewer retention, longer instructional videos, when structured into clearly defined and pedagogically coherent segments, are more effective for teaching complex subjects.^31^ This supports our observation that detailed procedural videos lead to higher learner engagement despite their extended duration.

### Limitations

Several limitations should be acknowledged in this study. First, although validated tools such as the ACS, GQS, mDISCERN, and JAMA were used to evaluate video quality, the subjective nature of these assessments may introduce potential bias, even with strong inter‐rater reliability. Second, the search was conducted on the global YouTube website, and the first 30 results per search term were analyzed. This search approach may not fully capture the complete range of available content, as country‐specific variations in YouTube can influence search results. Consequently, the availability of videos may differ based on geographic location, potentially excluding high educational value content in other languages or from different regions. Third, the analysis was restricted to English‐language content, which limits the generalizability of findings to non‐English educational contexts and excludes instructionally valuable resources in other languages. Future research should involve multilingual investigators to better explore educational resources in various languages. Fourth, the study did not assess learner performance or knowledge retention after viewing the videos, which would be essential to determine their true educational impact. Finally, our analysis revealed significant disparities in video coverage across anatomical regions. Most notably, the pelvis and perineum were represented by only one qualifying video compared to videos for other regions. This scarcity necessitated merging pelvic and perineal content with abdominal videos for quantitative analysis. This underscores the inadequacy of YouTube as a standalone resource for comprehensive anatomical education.

## CONCLUSION

In conclusion, this study critically assessed the quality of YouTube videos on surface anatomy, finding that 60% of the analyzed videos were educationally valuable, while popularity metrics (e.g., views and likes) did not reliably reflect the scientific quality. These results highlight the need for standardized, peer‐reviewed educational content on platforms like YouTube. To enhance resource quality, educators and institutions should prioritize clear tagging, comprehensive coverage, and best practices in video production, including training faculty in digital content creation. This framework empowers healthcare students to identify reliable resources and guides educators in creating effective, engaging anatomical videos.

## AUTHOR CONTRIBUTIONS

**Anas J. Mistareehi:** Conceptualization; methodology; writing – original draft; validation; visualization; investigation. **Ibrahim Hoja:** Conceptualization; methodology; visualization; formal analysis; writing – original draft; investigation. **Abdulrahman Alraddadi:** Validation; writing – review and editing. **Heba H. Ghozlan:** Visualization; writing – review and editing. **Ayman Mustafa:** Validation; writing – review and editing. **Mohammed Z. Allouh:** Project administration; funding acquisition; writing – original draft; writing – review and editing; resources; supervision.

## FUNDING INFORMATION

This work was supported by a UPAR grant (Grant code G00004977, Fund no. 12M219) and a SURE Plus grant (Grant code G00005273, Fund no. 12M280) to M. Z. Allouh from the United Arab Emirates University, UAE.

## CONFLICT OF INTEREST STATEMENT

The authors declare that they have no competing financial interests or personal relationships that could have appeared to influence the work reported in this article.

## ETHICS STATEMENT

This study exclusively utilized publicly available YouTube videos and did not involve any human participants, patient data, or animal subjects. Therefore, the research did not require approval by an institutional ethics committee in accordance with prevailing guidelines on the use of publicly sourced, non‐identifiable data. All data analyzed were in the public domain, and no individually identifiable information was recorded or used at any stage of this work.

## Supporting information


Data S1.

Table S1.
