# Supplementary material for: Evaluating the quality and educational utility of YouTube videos in teaching human surface anatomy
Source: Anat Sci Educ. 2025 Nov 20;19(3):440–51. doi: 10.1002/ase.70160 (PMC12996755; doi:10.1002/ase.70160)
Supplement: Supplementary file 1 — Data S1. [file ASE-19-440-s002.pdf]

	Video	Authour	Region	Search Ranking	Using Model	Uploade country	Time (sec)	Uploade Date	Days	Views	Like	Dislike	ACS	ACS Uesfulne	GQS	GQS Uesfulness	mDISCERN	JAMA
1	Surface anatomy landmarks of the head and neck (preview) - Human Anatomy | Kenhub	Kenhub - Learn Huma	Head & Neck	1	Drawings/images	Germany	210	January 31, 2019	2155	42003	411	56	12	No	3	No	3	2
2	Surface Anatomy of the Head	Clinical Examination	Head & Neck	1	Drawings/images	UK	435	October 9, 2018	2269	20022	240	0	15	Yes	5	Yes	5	3
3	Surface anatomy of head and neck part A	easy humananatomy	Head & Neck	1	Drawings/images	Bangladesh	507	September 18, 2023	464	663	5	0	16	Yes	5	Yes	5	1
4	Surface anatomy of Head Neck Face	Dr Pratimaa Dr Mukkt	Head & Neck	1	Living/cadaver	Unknown	552	July 9, 2021	1265	28945	419	16	13	No	4	Yes	4	1
5	Surface marking of Head & Neck | Dr Sibani Mazumder | Clinical Anatomy	Anatomy Simplified w	Head & Neck	1	Living/cadaver	India	794	May 23, 2024	216	2022	191	0	15	Yes	5	Yes	5	2
6	https://www.youtube.com/watch?v=ZCSmuwia00k&list=PL7ZT693bGivXj4x24ITPvkvXY4alxaTuw	EZ Anat	Head & Neck	2	Living/cadaver	Unknown	324	October 20, 2021	1162	38918	345	7	13	No	3	No	3	1
7	Surface Anatomy of the Neck	ጤናችን	Head & Neck	2	Drawings/images	Kenya	220	September 1, 2021	1211	1628	32	0	9	No	3	No	3	1
8	#firstyear #anat |surface anatomy of the head region by Dr.Akash Singh | head and Neck anatomy lec 1	JK Dent-Ease Dr.Jasm	Head & Neck	3	Drawings/images	India	1732	June 23, 2021	1281	2670	72	0	15	Yes	5	Yes	5	1
9	106.Surface Anatomy of Neck #anatomylectures	Dr Sujatha's ENT Clas	Head & Neck	3	Drawings/images	India	666	July 4, 2021	1270	5009	116	0	15	Yes	4	Yes	4	1
10	Surface Anatomy Head	Rita Thrasher	Head & Neck	3	Models	Unknown	147	April 12, 2020	1718	812	11	0	7	No	1	No	1	1
11	Living Anatomy & Head, Neck , Face	Pravara Institute Of M	Head & Neck	1	Drawings/images	India	3211	May 21, 2020	1679	3676	103	5	15	Yes	5	Yes	5	3
12	https://www.youtube.com/watch?v=IpOrJXeEzy8&list=PLr8DXQlPn2eGd34vo5p8rLf3sgUDes2I9	easy humananatomy	Head & Neck	2	Drawings/images	Bangladesh	3678	August 31, 2023	482	853	10	0	16	Yes	5	Yes	5	1
13	Anatomy - How to Draw the Dermatomes of the Arm, Head, and Neck	DrawItKnowIt	Head & Neck	3	Drawings/images	Unknown	219	August 3, 2016	3066	44228	637	13	11	No	3	No	3	1
14	Sensation of the face	The Noted Anatomist	Head & Neck	1	Drawings/images	USA	184	October 30, 2015	3344	78886	757	22	16	Yes	4	Yes	4	3
15	Nerve supply of skin of face,neck & scalp (sensory) | Anatomy	The Doc-tomy Girl	Head & Neck	1	Drawings/images	Unknown	262	June 12, 2020	1657	5967	116	0	13	No	4	Yes	4	1
16	Surface anatomy of the thorax	Human Anatomy Edu	Thorax	1	Living/cadaver	Canada	1092	September 21, 2011	4844	362893	5094	98	16	Yes	5	Yes	5	2
17	Surface Anatomy of the Thorax	More Than Skin Deep	Thorax	1	Drawings/images	UK	521	November 2, 2020	1514	39287	924	9	16	Yes	4	Yes	4	2
18	Surface anatomy landmarks of the thorax and upper limb (preview) - Human Anatomy | Kenhub	Kenhub - Learn Huma	Thorax	1	Drawings/images	Germany	213	January 29, 2019	2157	73843	906	80	13	No	2	No	2	2
19	Surface Anatomy of Landmarks of Thorax and Pleura	Viva Voce of Anatomy	Thorax	1	Living/cadaver	India	808	April 21, 2021	1344	68988	1146	26	12	No	3	No	3	2
20	Surface Anatomy - Pleura Lungs (2D)	RCSI	Thorax	1	Drawings/images	Ireland	420	December 14, 2015	3299	30289	324	13	16	Yes	5	Yes	5	4
21	BJMC: Surface Anatomy of Thorax	Anatomy Department	Thorax	2	Living/cadaver	Unknown	1170	May 4, 2021	1331	3047	75	0	15	Yes	4	Yes	4	1
22	Surface Marking of Thorax and it's clinical Importance	Anatomy Simplified w	Thorax	2	Living/cadaver	India	1028	July 18, 2022	891	16266	441	1	16	Yes	5	Yes	5	2
23	Surface anatomy of Chest /Thorax	Dr Pratimaa Dr Mukkt	Thorax	2	Living/cadaver	Unknown	362	June 11, 2021	1293	2309	68	0	13	No	3	No	3	1
24	Surface marking of lung	Emmanuel Bhaskar	Thorax	3	Drawings/images	India	190	July 26, 2016	3074	78067	929	31	16	Yes	4	Yes	4	1
25	Surface Anatomy of the Abdomen – Anatomy | Lecturio	Lecturio Medical	Abdomen	1	Drawings/images	USA	980	April 30, 2018	2431	187640	2116	70	16	Yes	5	Yes	5	3
26	Surface anatomy of the abdomen	Human Anatomy Edu	Abdomen	1	Living/cadaver	Canada	1502	February 5, 2012	4707	183255	2039	62	16	Yes	5	Yes	5	2
27	Surface Anatomy of Abdomen	Access Anatomy	Abdomen	1	Drawings/images	Unknown	252	May 7, 2014	3885	27732	195	6	16	Yes	3	No	3	2
28	Abdominal quadrants, regions, and planes	Osmosis from Elsevie	Abdomen	1	Drawings/images	USA	494	January 9, 2024	351	75507	1236	0	14	Yes	4	Yes	4	1
29	Surface marking of Abdomen	Anatomy Simplified w	Abdomen	1	Living/cadaver	India	1015	October 12, 2022	805	47807	880	4	16	Yes	5	Yes	5	2
30	9 and 4 abdominal anatomical regions	Sam Webster	Abdomen	1	Models	UK	728	September 9, 2021	1203	66746	1925	23	15	Yes	4	Yes	4	1
31	SURFACE LANDMARKS & SURFACE MARKINGS OF ABDOMEN	Anatomy Self Learnin	Abdomen	1	Living/cadaver	India	810	June 26, 2020	1643	65829	953	21	9	No	3	No	3	1
32	9 Regions of Abdomen made simple	MEDSimplified	Abdomen	2	Drawings/images	India	251	November 4, 2015	3339	491460	6665	269	13	No	4	Yes	4	1
33	Four Abdominal Quadrants and Nine Abdominal Regions - Anatomy and Physiology	RegisteredNurseRN	Abdomen	2	Drawings/images	USA	388	May 23, 2019	2043	398285	6991	116	15	Yes	3	No	3	1
34	Surface Anatomy of the Abdomen	Clinical Examination	Abdomen	2	Living/cadaver	UK	415	February 9, 2021	1415	2287	35	0	16	Yes	4	Yes	4	3
35	EXAMINE OF SURFACE MARKING OF PLANES AND REGIONS OF ABDOMEN	Dr C.kishan reddy	Abdomen	3	Living/cadaver	Unknown	369	February 14, 2024	315	1063133	3347	59	11	No	3	No	3	1
36	https://www.youtube.com/watch?v=7gW89r8PJtE&list=PLo9HUmd1d2zmWrKTEhQo6ysO6xN2gf0B8	Dr Hasna	Abdomen	3	Drawings/images	UK	604	March 2, 2023	664	2592	58	0	16	Yes	4	Yes	4	1
37	Bony abdomen landmarks (anatomy)	Sam Webster	Abdomen	1	Models	UK	842	September 16, 2021	1196	21356	684	2	16	Yes	4	Yes	4	1
38	Bony landmark of Abdomen	Dr.Jitendra Patel Anat	Abdomen	1	Living/cadaver	India	927	April 28, 2021	1337	779	22	0	14	No	3	No	3	1
39	Surface landmarks (bony landmarks) of anterior abdominal wall	Dr Chetana Sharma	Abdomen	2	Living/cadaver	Unknown	277	April 11, 2021	1354	728	19	0	14	No	3	No	3	2
40	Surface landmarks of abdomen | Surface anatomy of abdomen | Anatomy MCQ preparation | Dr. SAM	Dr. SAM's Anatomy Cl	Abdomen	2	Drawings/images	India	1255	July 11, 2021	1263	1409	72	0	15	Yes	4	Yes	4	1
41	ANTERIOR ABDOMINAL WALL - REGIONS OF ABDOMEN & IMPORTANT LANDMARKS WITH VERTEBRAL LEV	Learn with Dr Rajesh	Abdomen	3	Living/cadaver	Unknown	470	May 7, 2020	1693	6703	140	4	14	No	3	No	3	1
42	Landmarks of Abdomen ABDOMEN	Pragnyadaan Academ	Abdomen	3	Drawings/images	India	1661	August 25, 2020	1583	1197	21	0	14	No	4	Yes	4	1
43	Landmarks - Sacrum and Pelvic Region	Yein Lee	Pelviic	3	Living/cadaver	Unknown	178	January 10, 2019	2176	6611	85	0	13	No	3	No	3	2
44	Surface Anatomy of the Back	ጤናችን	Back	1	Drawings/images	Kenya	271	September 1, 2021	1211	3165	25	0	12	No	4	Yes	4	1
45	FINGER RULE METHOD: HOW TO PALPATE THORACIC SPINOUS AND TRANSVERSE PROCESSES?	Physio Classroom	Back	1	Living/cadaver	India	419	December 23, 2019	1829	129412	2693	24	16	Yes	4	Yes	4	2
46	Surface anatomy: Scapula, Posterior aspect	Chee-Wee Tan	Back	2	Living/cadaver	UK	82	September 4, 2013	4130	26023	162	5	12	No	2	No	2	1
47	Thoracic Spine Anatomy and Palpation with Michael Lucido	NAIOMT	Back	2	Living/cadaver	Unknown	166	April 19, 2016	3172	132510	698	75	13	No	3	No	3	2
48	Surface anatomy of Back (on mummified body)	Dr Pratimaa Dr Mukkt	Back	2	Living/cadaver	Unknown	249	March 31, 2022	1000	290	11	0	13	No	3	No	3	1
49	Surface anatomy of back | Lumbar puncture | Spinal tap | Spinal anaesthesia | Palpating the spine	Dr. SAM's Anatomy Cl	Back	3	Living/cadaver	India	214	June 22, 2021	1282	3718	82	0	16	Yes	4	Yes	4	1
50	Landmarks Of Back Of Body | | anatomy	Creative Medicine	Back	3	Drawings/images	India	299	July 21, 2015	3445	8593	141	7	10	No	3	No	3	1
51	Bony Landmarks of the Back	CotswoldAcademy	Back	2	Living/cadaver	UK	137	May 7, 2020	1693	459	12	0	16	Yes	4	Yes	4	1
52	Upper Limb: Surface Anatomy & Osteology – Anatomy | Lecturio	Lecturio Medical	Upper Limb	1	Drawings/images	USA	2976	May 14, 2018	2417	337684	5179	76	16	Yes	4	Yes	4	3
53	Surface Anatomy - Introduction, Upper and Lower Limbs	Clinical Examination	Upper Limb	1	Living/cadaver	UK	1606	October 23, 2018	2255	19319	296	0	16	Yes	5	Yes	5	3
54	Anatomy Made Easy: Surface Marking of the Upper Limb | #medicalstudents	Anatomy Simplified w	Upper Limb	1	Living/cadaver	India	736	April 6, 2023	629	7705	290	1	16	Yes	5	Yes	5	2
55	Surface Anatomy - Wrist Hand (2D)	RCSI	Upper Limb	1	Drawings/images	Ireland	721	December 14, 2015	3299	25103	353	6	16	Yes	5	Yes	5	3
56	https://www.youtube.com/watch?v=m8tkPnJcLpM&list=PLGnVyHLo4ymu6YgljUzN5y-y-Bwqljh8H	Chee-Wee Tan	Upper Limb	1	Living/cadaver	UK	951	September 5, 2013	4129	17532	60	4	16	Yes	5	Yes	5	1
57	surface marking of upper limb	Anatomy by Dr. Palak	Upper Limb	1	Living/cadaver	India	1040	April 16, 2021	1349	110294	2325	37	14	No	3	No	3	1
58	Surface Anatomy - Shoulder Girdle (2D)	RCSI	Upper Limb	1	Drawings/images	Ireland	1501	December 14, 2015	3299	7806	82	4	16	Yes	5	Yes	5	3
59	Upper limb surface anatomy	Dr Anshu's MBBS Lec	Upper Limb	2	Drawings/images	Unknown	197	March 3, 2018	2489	3276	38	0	14	No	4	Yes	4	1
60	https://www.youtube.com/watch?v=9CTMSEQXJ6E&list=PL7ZT693bGivUdjYR_KnMhC1y1ffqp_UXt	EZ Anat	Upper Limb	2	Living/cadaver	Unknown	295	October 20, 2021	1162	32960	289	7	13	No	3	No	3	1
61	Superficial Veins of Upper Limb - Basilic & Cephalic veins | Anatomy Tutorial	Anatomy Knowledge	Upper Limb	2	Drawings/images	Unknown	234	October 13, 2020	1534	266439	4798	100	14	No	4	Yes	4	1
62	Surface Anatomy of Upper limb Dr Shabana Lectures	Dr Shabana Anatomy	Upper Limb	2	Drawings/images	Unknown	2900	March 31, 2021	1365	3864	75	0	15	Yes	5	Yes	5	1
63	Surface Anatomy - The Arm (2D)	RCSI	Upper Limb	3	Drawings/images	Ireland	745	December 14, 2015	3299	10474	149	0	16	Yes	5	Yes	5	3
64	Bony Landmarks of the Body Palpation	Dr. Justin Lee, Doctor	Upper Limb	1	Living/cadaver	USA	888	May 25, 2020	1675	20900	777	19	15	Yes	4	Yes	4	1
65	Bony Landmark Palpation - Upper Extremity (Clavicle, Scapula, Humerus, Radius, Ulna, Carpals)	Blackriver & Bootsma	Upper Limb	1	Living/cadaver	Canada	5650	November 15, 2022	771	115957	493	16	16	Yes	5	Yes	5	1

	Video	Authour	Region	Search Ranking	Using Model	Uploade country	Time (sec)	Uploade Date	Days	Views	Like	Dislike	ACS	ACS Uesfulne	GQS	GQS Uesfulness	mDISCERN	JAMA
66	Upper Limb Dermatomes | Clinical Physio	Clinical Physio	Upper Limb	1	Living/cadaver	UK	910	October 30, 2019	1883	94909	1929	21	16	Yes	5	Yes	5	1
67	Upper limb dermatomes	Sam Webster	Upper Limb	1	Models	UK	784	October 6, 2022	811	25070	869	0	16	Yes	4	Yes	4	1
68	Dermatomes and Pure Patches: upper extremity	NeuroDocUniverse	Upper Limb	1	Drawings/images	Unknown	443	June 16, 2011	4941	34732	225	9	13	No	2	No	2	1
69	17. Dermatomes of Upper Limb Anatomy : MBBS Lecture	Med School Simplifie	Upper Limb	1	Drawings/images	India	927	June 23, 2024	185	1478	27	0	16	Yes	4	Yes	4	1
70	Trick to remember permanently Dermatomes of upper limb	OxyMBBS	Upper Limb	2	Drawings/images	India	913	February 12, 2018	2508	64703	1080	82	14	No	3	No	3	1
71	Lower Limb: Surface Anatomy & Osteology – Anatomy | Lecturio	Lecturio Medical	Lower Limb	1	Drawings/images	USA	2078	November 9, 2020	1507	135237	1842	56	16	Yes	4	Yes	4	3
72	https://www.youtube.com/watch?v=jjeOx7rYz4Q&list=PLGnVyHLo4ymuK-rZsP5WwLkEOJpyufFJB	Chee-Wee Tan	Lower Limb	1	Living/cadaver	UK	265	July 18, 2014	3813	92873	353	14	15	Yes	4	Yes	4	1
73	https://www.youtube.com/watch?v=ojkYPvj4C0w&list=PL7ZT693bGivWTVRJ_k4L-m_U5lH0xbyO	EZ Anat	Lower Limb	1	Living/cadaver	Unknown	400	July 18, 2014	3813	26963	289	2	13	No	3	No	3	1
74	Surface Marking of Lower Limb	Chelsey Bansal	Lower Limb	1	Living/cadaver	Unknown	831	March 20, 2021	1376	92931	2004	60	14	No	3	No	3	1
75	LOWER LIMB -SURFACE MARKING(CLINICAL ANATOMY)	Anatomy Simplified w	Lower Limb	1	Living/cadaver	India	672	August 26, 2022	852	2855	93	0	16	Yes	5	Yes	5	1
76	Surface Anatomy of (Infex)Lower limb (Inferior Extremity)	Dr Pratimaa Dr Mukkt	Lower Limb	2	Living/cadaver	Unknown	516	March 26, 2022	1005	2031	30	0	13	No	3	No	3	1
77	Lower Limb Surface Anatomy | Professor Stuart Enoch	Doctors Academy	Lower Limb	2	Living/cadaver	Unknown	208	April 29, 2020	1701	7942	131	3	15	Yes	4	Yes	4	2
78	Surface Anatomy - Leg & Foot (2D)	RCSI	Lower Limb	2	Drawings/images	Ireland	346	December 14, 2015	3299	10971	107	2	16	Yes	5	Yes	5	3
79	Landmarks of the Lower Extremity	Med School Made Eas	Lower Limb	3	Living/cadaver	USA	132	July 23, 2013	4173	6147	75	2	12	No	3	No	3	1
80	ANARTOMY: Surface Anatomy of the Posterior Lower Limb	Anartomy	Lower Limb	3	Drawings/images	Unknown	592	March 24, 2020	1737	339	5	0	16	Yes	4	Yes	4	1
81	ANARTOMY: Surface Anatomy of the Anterior Lower Limb	Anartomy	Lower Limb	3	Drawings/images	Unknown	841	March 24, 2020	1737	648	14	0	16	Yes	4	Yes	4	1
82	Lower Limb Dermatomes | Clinical Physio	Clinical Physio	Lower Limb	1	Living/cadaver	UK	906	March 2, 2020	1759	65702	1062	8	16	Yes	5	Yes	5	1
83	Anatomy - Dermatomes of the Lower Extremity	DrawItKnowIt	Lower Limb	1	Drawings/images	Unknown	171	October 24, 2017	2619	60130	551	9	15	Yes	4	Yes	4	1
84	Dermatomes of Lower Limb.	Anatomy Stuff by Dr F	Lower Limb	1	Drawings/images	Pakistan	613	July 20, 2020	1619	7618	95	0	14	No	3	No	3	1
85	Dermatomes of lower limb made easy , in 5 min , simple , memorize	Muhammad Siraj	Lower Limb	2	Drawings/images	Unknown	310	July 14, 2020	1625	1307	17	0	14	Yes	3	No	3	1
