# Supplementary material for: Evaluating the quality and educational utility of YouTube videos in teaching human surface anatomy
Source: Anat Sci Educ. 2025 Nov 20;19(3):440–51. doi: 10.1002/ase.70160 (PMC12996755; doi:10.1002/ase.70160)
Supplement: Supplementary file 2 — Table S1. [file ASE-19-440-s001.docx]

**Supplementary Tables

Table S1: Global Quality Scale (GQS) scoring criteria**

| 1. Poor quality, poor flow, most information missing, not helpful for education |
| --- |
| 2. Generally, poor, some information given, but of limited use to education |
| 3. Moderate quality, some important information is adequately discussed |
| 4. Good quality, good flow, most relevant information is covered, useful for education |
| 5. Excellent quality and excellent flow, very useful for education |

The 5-point GQS was used to assess video educational quality, ranging from 1 (Poor) to 5 (Excellent).

**Table S2: Modified DISCERN (mDISCERN) scoring criteria for information reliability**

| Are the aims clear and achieved? | No: 0 Yes: 1 |
| --- | --- |
| - Are reliable sources of information used? | No: 0 Yes: 1 |
| - Is the information presented both balanced and unbiased? | No: 0 Yes: 1 |
| - Are additional sources of information listed for patient reference? | No: 0 Yes: 1 |
| - Are areas of uncertainty mentioned? | No: 0 Yes: 1 |

Each criterion was scored dichotomously (0=absent, 1=present).

**Table S3: Journal of the American Medical Association (JAMA) scoring criteria for source transparency and accountability**

| - **Authorship**: Authors and contributors, their affiliations, and relevant credentials should be provided | No: 0 Yes: 1 |
| --- | --- |
| - **Attribution**: References and sources for all content should be listed clearly, and all relevant copyright information should be noted | No: 0 Yes: 1 |
| - **Disclosure**: Website “ownership” should be prominently and fully disclosed, as should any sponsorship, advertising, underwriting, commercial funding arrangements or support, or potential conflicts of interest | No: 0 Yes: 1 |
| - **Currency**: Dates when content was posted and updated should be indicated | No: 0 Yes: 1 |

Each criterion was scored dichotomously (0=absent, 1=present)
